# Genome-wide characterization, evolution, and expression analysis of the leucine-rich repeat receptor-like protein kinase (LRR-RLK) gene family in Rosaceae genomes

**DOI:** 10.1186/s12864-017-4155-y

**Published:** 2017-10-10

**Authors:** Jiangmei Sun, Leiting Li, Peng Wang, Shaoling Zhang, Juyou Wu

**Affiliations:** Centre of Pear Engineering Technology Research, State Key Laboratory of Crop Genetics and Germplasm Enhancement, Nanjing Agricultural University, Nanjing, 210095 China

**Keywords:** Rosaceae, Leucine-rich repeat receptor-like protein kinase (LRR-RLK), Tandem duplication, Gene expression

## Abstract

**Background:**

Leucine-rich repeat receptor-like protein kinase (LRR-RLK) is the largest gene family of receptor-like protein kinases (RLKs) and actively participates in regulating the growth, development, signal transduction, immunity, and stress responses of plants. However, the patterns of LRR-RLK gene family evolution in the five main Rosaceae species for which genome sequences are available have not yet been reported. In this study, we performed a comprehensive analysis of LRR-RLK genes for five Rosaceae species: *Fragaria vesca* (strawberry), *Malus domestica* (apple), *Pyrus bretschneideri* (Chinese white pear), *Prunus mume* (mei), and *Prunus persica* (peach), which contained 201, 244, 427, 267, and 258 LRR-RLK genes, respectively.

**Results:**

All LRR-RLK genes were further grouped into 23 subfamilies based on the hidden Markov models approach. RLK-Pelle_LRR-XII-1, RLK-Pelle_LRR-XI-1, and RLK-Pelle_LRR-III were the three largest subfamilies. Synteny analysis indicated that there were 236 tandem duplicated genes in the five Rosaceae species, among which subfamilies XII-1 (82 genes) and XI-1 (80 genes) comprised 68.6%.

**Conclusions:**

Our results indicate that tandem duplication made a large contribution to the expansion of the subfamilies. The gene expression, tissue-specific expression, and subcellular localization data revealed that LRR-RLK genes were differentially expressed in various organs and tissues, and the largest subfamily XI-1 was highly expressed in all five Rosaceae species, suggesting that LRR-RLKs play important roles in each stage of plant growth and development. Taken together, our results provide an overview of the LRR-RLK family in Rosaceae genomes and the basis for further functional studies.

**Electronic supplementary material:**

The online version of this article (10.1186/s12864-017-4155-y) contains supplementary material, which is available to authorized users.

## Background

During growth and development, plants are affected by many different kinds of signal stimulation from cell-cell and cell-environment interactions, which trigger a series of signal transductions. The regulatory mechanism of reversible phosphorylation that involves protein kinases (PKs) plays an important role in cellular signal transduction processes and all living cells can perceive and process external or internal signals via cell surface PKs, which mostly belong to the receptor-like kinase (RLK) family [1, 2]. RLKs are ubiquitous in the plant kingdom. Since the first leucine-rich repeat receptor-like kinase (LRR-RLK) gene was isolated in maize (*Zea mays*) [3], more and more LRR-RLK genes have been confirmed [2, 4]. However, the function of most RLKs has not been confirmed and the natural ligands of most receptor proteins have not been identified.

LRR-RLK is the largest and most highly conserved group of the plant RLK gene family [5]. Previous studies have shown that the approximately 225 LRR-RLK gene members in *Arabidopsis* can be grouped into 23 subfamilies based on the sequence similarity in their kinase domains [2, 6, 7]. A typical LRR-RLK contains an extracellular tandem arrayed leucine-rich repeat domain, a single-pass transmembrane domain, and an intracellular functional protein kinase domain. The major class of extracellular domain is 20–30 amino acids long. With regards to tertiary structure, each LRR domain forms an α/ß helix and is located on the surface of the protein, enabling it to participate in protein-protein interactions [2, 8]. Because of their structural characteristics, LRR-RLKs play essential roles in mediating cell signal transduction pathways and critically control plant growth, development, responses to stresses, and immunity. In the Brassinosteroid (BR) and abscisic acid (ABA) signal pathways, LRR-RLKs play a pivotal role. BRs are important regulatory substances in plant development. The receptor kinase Brassinosteroid-insensitive 1 (BRI1), which has 25 LRR tandem structures in its extracellular portion, can perceive and deliver BR signaling [9]. BRI1-associated kinase 1 (BAK1) is another LRR-RLK that has five LRR tandem structures in its extracellular portion. The cross-talk between BRI1 and BAK1 makes up a dimer, the key step in the BR signal transduction [10]. In *Arabidopsis*, receptor-like protein kinase 1 (RPK1) is an LRR-RLK on the surface of the plasma membrane, the LRR domain of which is key to regulating ABA sensitivity [11]. Previous research has shown that the *rpk1* mutant exhibits decreased sensitivity to ABA-induced senescence in *Arabidopsis* leaves, but does not exhibit a change in jasmonic acid- and ethylene-induced senescence [12]. BAK1 not only plays a role in the process of BR signal transmission, but also forms a dimer with the resistance gene flagellin-sensitive 2 (FLS2), and is involved in plant immune responses. BAK1 may also combine with other ligands and participate in other LRR-RLK signaling pathways [13].

There are a large number of LRR-RLKs in plants, but intensive studies on their functions have only been performed for a few genes. Because of the complexity of plant signal transduction pathways and complementary functions between different proteins, very few LRR-RLK ligands and complex LRR-RLK signaling pathways have been found. Thus, there is a great need for system analyses of the evolution and expression differentiation of the LRR-RLK gene family using bioinformatics tools, as well as to identify genes that have potential functions in plant responses to environmental factors; this will also help us to improve our understanding of LRR-RLK functions in regulating networks during plant growth. Recently, LRR-RLK gene family have been analyzed in soybean [14], two *Citrus* species [15], *Amborella trichopoda* [16], and evolutionary studies in various species [17, 18]. Specifically, Fischer et al. investigated the involvement of selection during the expansion of the LRR-RLK gene family among angiosperms by analyzing the phylogeny of 7554 LRR-RLK genes from 31 fully sequenced flowering plant genomes, including two genomes of Rosaceae, apple (*Malus domestica*) and peach (*Prunus persica*) [18]. However, a more comprehensive investigation of LRR-RLK genes in a wider range of Rosaceae genomes is needed. In this study, we aimed to elucidate the structure, expression, and evolution of Rosaceae LRR-RLK genes. To this end, we analyzed a total of 1622 LRR-RLK genes in 23 LRR-RLK subfamilies in five Rosaceae species [strawberry (*Fragaria vesca*), peach, mei (*Prunus mume*), apple and Chinese white pear (*Pyrus bretschneideri*)] and *Arabidopsis*, including their chromosomal location, gene structure, duplication events, expansion, expression, and co-expression relationships, which should provide genome-level insights on LRR-RLK genes.

## Results

### Genome-wide identification and classification of LRR-RLK genes

**Fig. 1:**
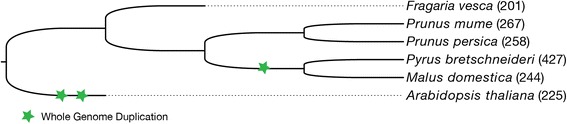
The phylogenetic tree of the species used for identifying LRR-RLK genes with whole genome duplication event marked in green star. The number in the parentheses indicate number of LRR-RLK genes in each species

We searched all annotated genes in the five Rosaceae species (strawberry, peach, mei, apple and Chinese white pear as shown in Table 1 and Fig. 1) for putative PKs and identified 6680 typical PKs with the number of typical PKs in each species ranged from 856 in strawberry to 1614 in Chinese white pear (Additional file 1: Tables S1 and S2). PKs were then classified into families and subfamilies using the HMM search approach, as described in the Methods section, which resulted in 1622 genes being classified into the LRR-RLK family, ranging from 201 genes in strawberry to 427 genes in Chinese white pear (Fig. 1, Table 2, Additional file 1: Table S3). The LRR-RLK genes identified in *Arabidopsis* and Rosaceae were further classified into 23 subfamilies and showed different patterns of gene subfamily size. Of these subfamilies, XII-1 (also named RLK-Pelle_LRR-XII-1), XI-1, and III were the top three largest subfamilies with an average number of members of 47.2, 66.0, and 44.0. The other subfamilies consisted of no more than 24 members, of which Xb-2 was the smallest subfamily with only one or two members.

**Table 1:** Genome information for *Arabidopsis* and five sequenced Rosaceae species

Species name	Common name	Release version*	Gene number	Gene ID
*Arabidopsis thaliana*	*Arabidopsis*	TAIR 10	27,416	AT-, At-
*Fragaria vesca*	Strawberry	GDR, v1.1	34,809	mrna-, Fve-
*Malus domestica*	Apple	GDR, v1.0 primary	30,294	MDP-, Mdo-
*Pyrus bretschneideri*	Chinese white pear	NJAU, v1.0	42,812	Pbr-
*Prunus mume*	Mei	BJFU, v1.0	31,390	Pm-, Pmu-
*Prunus persica*	Peach	JGI, v1.0	27,852	ppa-, Ppe-

* *TAIR* The *Arabidopsis* Information Resource, *NJAU* Nanjing Agricultural University, *GDR* Genome Database for Rosaceae, *BJFU* Beijing Forestry University, and *JGI* Joint Genome Institute

**Table 2:** List of 23 LRR-RLK subfamily names and numbers of *Arabidopsis* and Rosaceae

LRR-RLK subfamily	*Arabidopsis*	Strawberry	Apple	Chinese white pear	Mei	Peach	Total
I-1	48	15	15	17	22	24	155
I-2	2	3	3	3	3	3	22
II	14	8	11	14	12	11	90
III	46	36	46	62	37	37	337
IV	3	3	2	6	3	3	25
IX	4	6	6	11	6	6	49
V	9	7	7	10	6	6	55
VI-1	5	5	6	7	5	5	44
VI-2	8	4	5	4	4	4	36
VII-1	5	4	6	7	4	4	37
VII-2	3	2	2	4	2	2	19
VII-3	2	2	4	4	3	2	21
VIII-1	8	9	3	12	8	10	60
Xa	4	4	5	4	3	3	30
Xb-1	9	9	11	16	9	9	82
Xb-2	1	1	1	2	1	1	9
XI-1	33	56	64	105	72	66	492
XI-2	2	1	4	6	2	2	21
XII-1	8	18	35	115	56	51	376
XIIIa	4	3	2	4	3	3	25
XIIIb	3	2	3	4	2	2	19
XIV	2	2	2	5	2	2	19
XV	2	1	1	5	2	2	18
Grand Total	225	201	244	427	267	258	1622

**Table 3:** Statistical analysis of intron number across different subfamilies

LRR-RLK Subfamily	Number of genes	Minimum number of introns	Maximum number of introns	Average number of introns*	Standard deviation
XIIIb	16	22	42	27.2^aA^	4.20
VIII-1	50	4	32	16.6^bB^	5.24
V	45	4	35	15.9^bB^	5.00
I-1	141	1	26	11.3^cC^	3.85
XIIIa	19	4	20	10.6c^dC^	4.49
VI-2	29	4	13	9.4^dC^	2.72
II	70	2	20	9.4^dC^	2.79
VI-1	33	4	12	6.6^eD^	1.50
I-2	17	5	10	6.3^eD^	1.10
XIV	15	3	6	3.5^fE^	0.92
IV	20	3	6	3.4f^gE^	0.88
XII-1	283	0	14	2.6^fghEF^	2.26
VII-2	15	0	16	2.1^fghiEFG^	3.97
XI-2	17	0	22	2.0^fghiEFG^	5.23
III	264	0	18	1.8^ghijEFG^	2.08
XI-1	396	0	11	1.7^hijEFG^	1.48
IX	39	1	5	1.2^hijFG^	0.68
VII-1	30	1	2	1.1^hijFG^	0.25
Xb-2	7	0	2	0.9^ijFG^	0.69
Xb-1	63	0	10	0.7^ijFG^	1.69
XV	13	0	7	0.6^ijFG^	1.94
Xa	23	0	2	0.3^jG^	0.56
VII-3	17	0	1	0.2^jG^	0.44

* Significant difference analysis was performed by Duncan’s test. Lower letters indicate significant difference (*P* < 0.05); upper letters indicate extreme significant difference (*P* < 0.01)

**Fig. 2:**
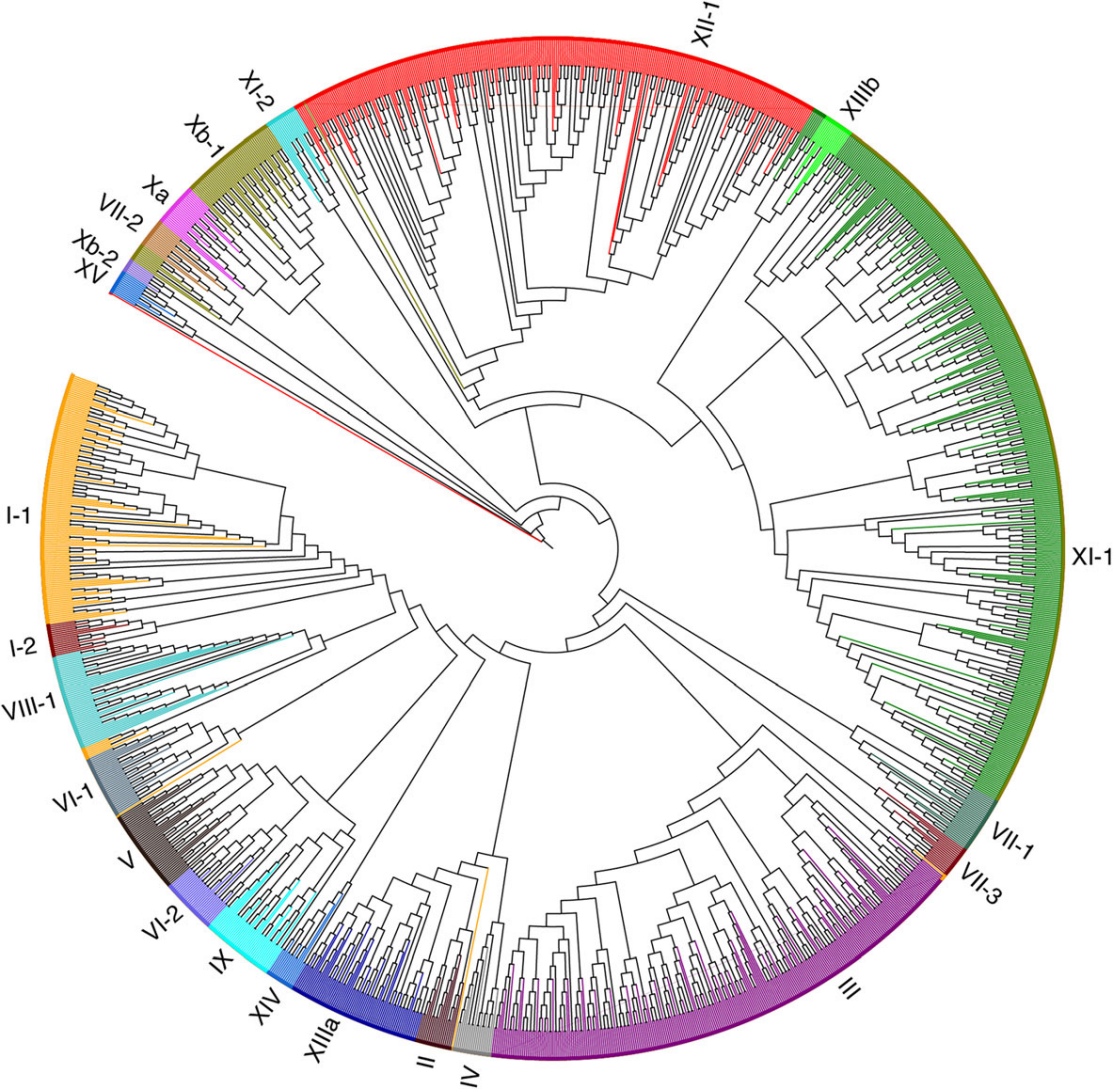
The phylogenetic tree for LRR-RLK genes identified in *Arabidopsis* and Rosaceae. A total of 1622 LRR-RLK genes were classified into 23 subfamilies and are distinguished by different colors

To confirm the accuracy of identification results, we used the method above to identify the LRR-RLK genes in *Arabidopsis* as a reference (Additional file 1: Tables S1 and S2) and compared the identified LRR-RLK genes in *Arabidopsis* in this study with previously reported results [6]. The list of LRR-RLK genes in *Arabidopsis* in this study was identical as reported previously [6], indicating that our gene identification procedure was reliable. We also constructed a phylogenetic tree for LRR-RLK genes in *Arabidopsis* and the five Rosaceae species (Fig. 2), which showed that most genes in the same subfamily were classified together.

### Protein domain and intron number analyses revealed divergent evolution for different subfamilies

The total of 1622 LRR-RLK genes identified from *Arabidopsis* and the five Rosaceae species were scanned against the Pfam database version 28.0 to identify additional protein domains besides Pkinase that may be common among subfamilies. The protein domains identified for each gene are listed in Additional file 1: Table S4. The basic composition of the LRR-RLK gene family consisted of a PK domain and an LRR domain; however, the results revealed that the frequency of Pkinase and LRR domains in each subfamily were different, and the preferred Pkinase and LRR domains were different (Additional file 1: Table S5). For instance, II preferentially included Pkinase, VI-1 preferentially included Pkinase_Tyr, while the result for VII-1 was mixed. In addition, XIIIa preferentially included LRRNT_2 (leucine-rich repeat N-terminal 2) and XIIIb preferentially included LRR_8 (leucine-rich repeat 8). And most genes have more than one type of LRR. These results suggest that different subfamilies have different compositions of protein domains, but genes in the same subfamily share common arrangements, indicating the common evolutionary history within the subfamilies.

**Fig. 3:**
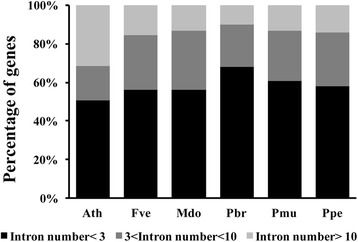
The frequency of genes with different intron sizes in the LRR-RLK gene family in *Arabidopsis*, strawberry, apple, Chinese white pear, mei, and peach

To obtain further insights into the structural diversity of the *Arabidopsis* and Rosaceae LRR-RLK genes, we analyzed their intron numbers (Table 3; Additional file 1: Table S6). These were found to vary widely, ranging from 0 to 42 (an LRR-RLK gene of mei, Pm019437). Most genes (965 or 59.5%, including 114 *Arabidopsis* genes) contained fewer than three introns (see Additional file 1: Table S6 for a summary and Additional file 1: Table S3 for a detailed list) and there were 250 genes with more than ten introns. Based on the frequency of LRR-RLK genes with different intron sizes (Fig. 3), we found that the frequency of Rosaceae genes with fewer than three introns was greater than that in *Arabidopsis*; in the latter, this proportion was 50%, compared with proportions ranging from 56% in apple to 68% in Chinese white pear. Similarly, the frequency of genes with an intron number between three and ten was higher in the five Rosaceae species than that in *Arabidopsis*. The average number of intron numbers in XIIIb was 27.2, significantly higher than other subfamilies. The average number of intron numbers in Xb-2, Xb-1, XV, Xa, VII-3 was lower than 1. These findings suggest that different subfamilies have different patterns of intron number and intron number could be used to represent the course of evolution in these subfamilies, and also verify our previous classification process.

### Segmental duplication

**Fig. 4:**
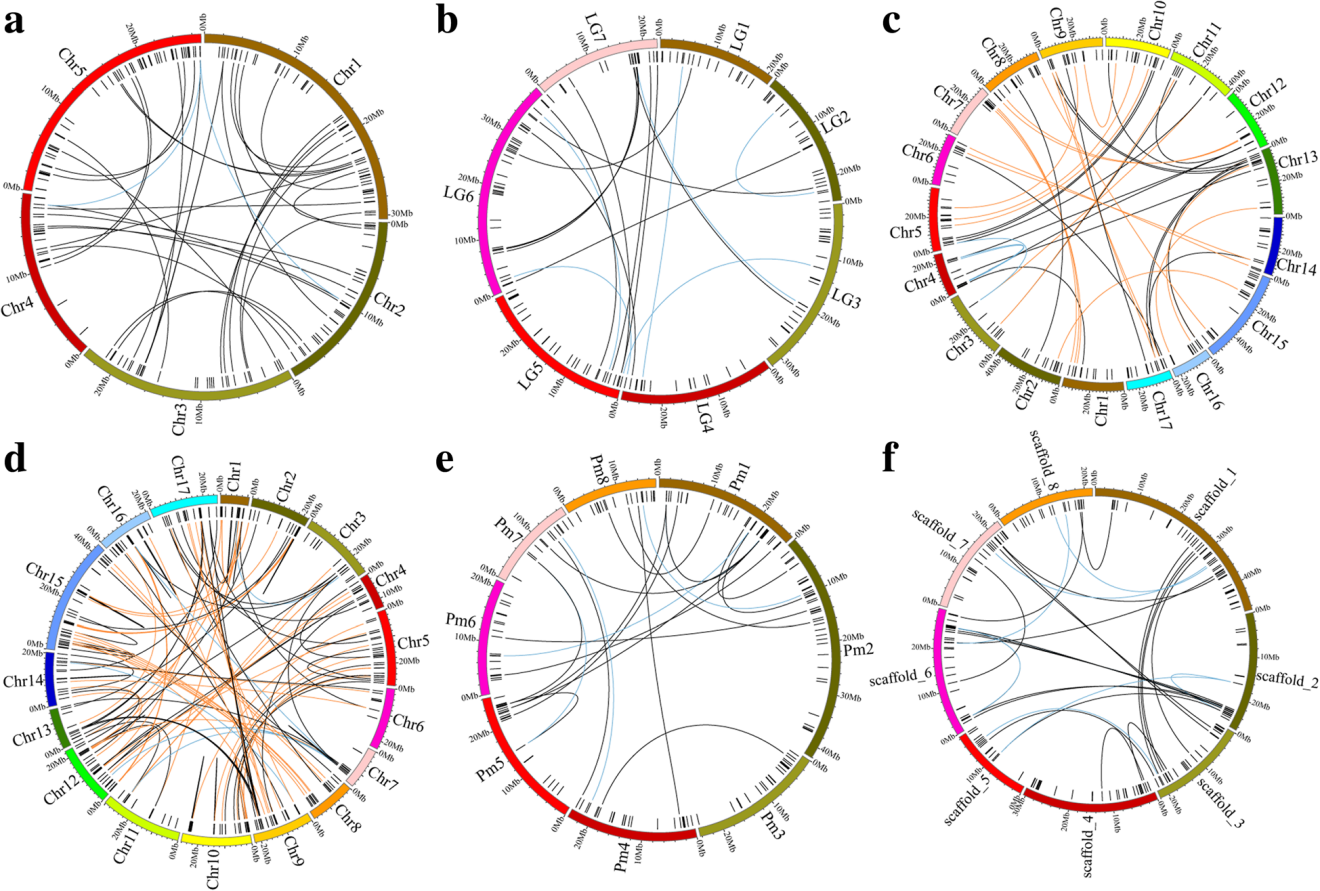
The circos figure for chromosome locations with segmental duplication links. **a** *Arabidopsis thaliana*, **b** *Fragaria vesca*, **c** *Malus domestica*, **d** *Pyrus bretschneideri*, **e** *Prunus mume*, **f** *Prunus persica*. The blue lines indicate segmented duplicated gene pairs that were traced to the paleohexaploidization event (~140 million years ago). The yellow lines indicate segmented duplicated gene pairs that were tranced to recent duplication event (30~45 million years ago)

Genome duplications are believed to have had a significant impact on the expansion of certain gene families in plants [19]. A previous study [20] suggested that the apple and Chinese white pear genomes had expanded through two rounds of duplication, named recent duplication and paleohexaploidization. The recent duplication occurred approximately 30–45 million years ago (MYA) [synonymous substitution rate (K_s_) between 0.15 and 0.30] in the apple and Chinese white pear genomes, while the paleohexaploidization occurred approximately 140 MYA (K_s_ between 1.5 and 1.8). To trace which genes originated from these two duplication events, intra-genome scanning of collinearity blocks was performed (Additional file 1: Table S7, Fig. 4) and K_s_ values were calculated for each gene pair. Gene pairs traced to the recent duplication were only found in apple and Chinese white pear, while gene pairs traced to the paleohexaploidization were found in all five Rosaceae species. However, considering a large number of genes of the LRR-RLK gene family in each species, only a few gene pairs were traced to the paleohexaploidization (*Arabidopsis thaliana*: 2, *Malus domestica*: 3, *Fragaria vesca*: 6, Pbr: 7, *Prunus mume*: 6, *Prunus persica*: 8). Among these gene pairs that were traced to the paleohexaploidization event, III was the only subfamily found in all five Rosaceae species, indicating that this subfamily is essential for the Rosaceae, given that it is ancient and conserved. On the other hand, 20 gene pairs in apple and 68 pairs in Chinese white pear were found to be associated with the recent duplication event, indicating that this event contributed more to the expansion of the LRR-RLK gene family in these two species. The proportion of LRR-RLK genes in each species associated with whole genome duplication (WGD)/segmental duplication (Additional file 1: Table S8) had a wide range, from 16% in mei to 51% in Chinese white pear. Although duplicated gene pairs could be found in all subfamilies, some of them were lineage-specific. For instance, XIIIa and VII-2 showed WGD/segmental duplication only in Chinese white pear, Xb-1 showed it only in apple and Chinese white pear, and XIV showed it only in Rosaceae, but not in *Arabidopsis*; these findings provide clues for how different subfamilies evolved in Rosaceae.

### Tandem duplication

Tandem duplication is another source of gene family expansion as it increases gene numbers and genome complexity [21–24]. Thus, to examine how tandem duplication contributed to LRR-RLK gene family expansion, we compared the number of tandem duplicated genes in each species (Additional file 1: Table S9) and found that there were 12 tandem arrays in *Arabidopsis*, 11 in strawberry, 14 in apple, 33 in Chinese white pear, 25 in mei, 27 in peach, and 37 tandem duplicated genes in *Arabidopsis*. These involved 24 genes in strawberry, 32 genes in apple, 67 genes in Chinese white pear, 51 genes in mei, and 62 genes in peach (Additional file 1: Table S10). In *Arabidopsis*, we found that most tandem duplication events (28/37 genes) had occurred in the RLK-Pelle_LRR-I-1 subfamily. Similarly, tandem duplications in strawberry, apple, Chinese white pear, mei, and peach seem to have contributed to the generation of approximately 50.0% (12/24 genes), 50.0% (16/32 genes), 25.4% (17/67 genes), 29.4% (15/51 genes), and 32.3% (20/62 genes) of the XI-1 subfamily, respectively (Additional file 1: Table S10). The XII-1 subfamily was also shown to have many genes generated through tandem duplications in Chinese white pear, mei, and peach.

### Expression pattern analysis by transcriptome data

To explore the expression patterns of LRR-RLK genes, we analyzed publicly available RNA-seq (RNA sequencing) or transcriptome data from the NCBI (National Center for Biotechnology Information) SRA (Sequence Read Archive) database, for a total of 108 samples from 18 experiments, including pooled organs, biotic stress, abiotic stress, tissue-specific expression, fruit development, and developmental biology (Additional file 1: Tables S11–S15, Additional file 2: Figures S1–S5). In general, we found that not all genes were expressed. The number of LRR-RLK genes in any tissue with FPKM >1 was 97 in strawberry (45.3%), 216 (88.5%) for apple, 338 (79.2%) for pear, 195 (73.0%) for mei and 58 (22.5%) for peach. These expressed (FPKM >1 in any tissue) genes were found in all LRR-RLK subfamilies.

**Fig. 5:**
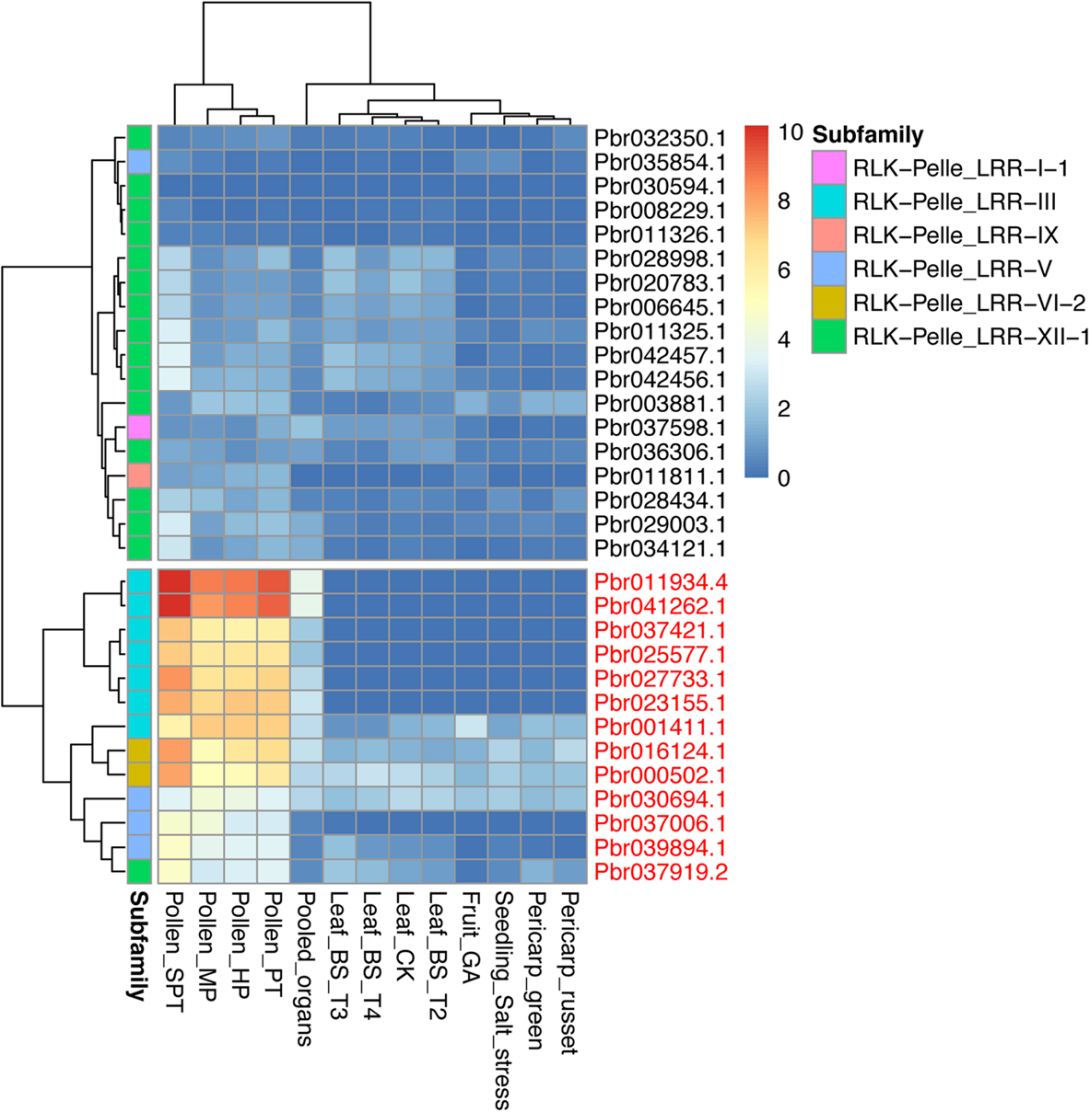
The heatmap of the expression of a cluster of pear genes that are highly expressed in pear pollens. The ID of the 13 genes that highly expressed in pollens are marked in red. The FPKM values are log2 transformed

**Fig. 6:**
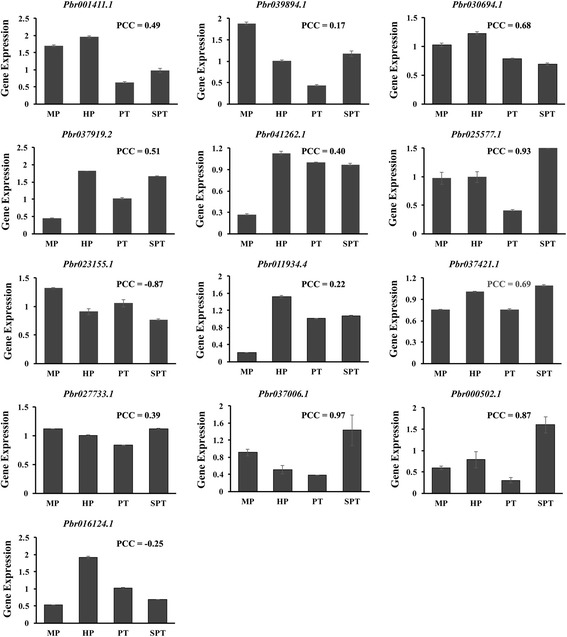
qRT-PCR verification of 13 pear genes in four developmental stages of pear pollens, including mature pollen grain (MP), hydrated pollen grain (HP), growing pollen tube (PT), and stopped-growth pollen tube (SPT). The Pearson Correlation Coefficient (PCC) of the expression patterns with their corresponding transcriptome data as presented in Fig. 5 are labeled for each gene

The expression profiles of genes in pollen, pistil, or stigma are an important issue in developmental biology. In Chinese white pear, the expression of LRR-RLK genes was examined in four growth stages of pollen: mature pollen grain (MP), hydrated pollen grain (HP), growing pollen tube (PT), and stopped-growth pollen tube (SPT) [25]. We found that a cluster of genes was relatively higher expressed in pear pollen (Fig. 5), among which 13 genes (names highlighted in red in Fig. 5) were relative higher expressed during pollen growth. These genes distributed in subfamilies III, V, VI-2, and XII-1 (Additional file 2: Figure S3, Additional file 1: Table S13). To confirm the expression patterns, we verified the expression by qRT-PCR as shown in Fig. 6. The results showed that 10 of these genes had similar expression patterns (Pearson correlation coefficiency (PCC) > 0.2, as labeled in Fig. 6) as transcriptomic data. Specifically, the top expressed gene Pbr011934.4 (subfamily III) was an ortholog of *Arabidopsis* gene *PRK1* (AT5G35390), which accumulated in the plasma membrane of the apical growing tip of pollen tube through the process of exocytosis [26]. Similarly, the subfamily III was also found highly expressed in the pollen of mei. But in the pistil of mei, the highly expressed genes distributed in a variety of subfamilies, including subfamily Xa, II, VI-2, XI-1, etc. One of the top highly expressed genes in the pistil of mei was Pm00409.1 (Subfamily II), an ortholog of *AtSERK2* (AT1G34210). And in the stigma of peach, subfamilies XI-1 and Xb-1 were highly expressed. The highest expressed gene in the stigma of peach was ppa001010m, a homolog of *Arabidopsis* gene AT1G09970.2 (Additional file 1: Table S15), which is involved in the control of germination speed and tolerance to oxidative stress [27]. These indicate spatial expression differences of LRR-RLK gene family in the development of flower.

Strawberry and apple are important fruit-producing crops, although the edible fruit in strawberry is different from apple in that it is actually enlarged receptacle tissue [28]. Expression in the apple mature fruit and developmental stages of strawberry fruits revealed that LRR-RLK genes were also involved in fruit development. Among these genes, the top three expressed genes in strawberry were mrna21119.1, mrna17482.1 and mrna05604.1, belonging to subfamilies Xb-1, Xa, and XI-1, respectively. These genes are orthologs of the *Arabidopsis* genes *AtBRI1*, *BIR1* and *HSL2* (Additional file 1: Table S11, Additional file 2: Figure S1). In apple mature fruits, the top three expressed genes in apple mature fruits were MDP0000148501, MDP0000280908 and MDP0000266980, belonging to the subfamilies XI-1, III and XIIIa, respectively, are orthologs of *Arabidopsis* genes *BAM1*, *RKL1* and *FEI1* (Additional file 1: Table S12, Additional file 2: Figure S2).

Additionally, LRR-RLK genes are also involved in the response to biotic and abiotic stresses. The top three highly expressed genes in the Chinese white pear samples that were treated with *Alternaria alternata* (black spot), salt and GA were Pbr024019.1 (subfamily XI-2), Pbr018826.1 (subfamily XI-1) and Pbr019916.1 (subfamily XIIIb), respectively. Furthermore, Pbr024019.1 is the ortholog of *Arabidopsis* gene *SOBIR* gene (AT2G31880.1), which is expressed in response to the bacteria *Pseudomonas syringae* and regulates cell death and innate immunity [29]. This indicates the potential roles in stress response of these top expressed genes.

### Co-expression network of LRR-RLK subfamilies

The gene expression patterns analyzed above showed that some genes have potential co-expression relationships. Thus, we created co-expression networks for LRR-RLK genes in each species using the available datasets as mentioned above. To construct these co-expression networks, we performed pairwise Pearson correlation coefficient (PCC) analyses for each species and used a threshold of PCC > 0.95 (Additional file 2: Figures S6–S10, Additional file 1: Table S16). In strawberry, ten LRR-RLK genes formed four small sub-network with two or three nodes. In apple, 66 LRR-RLK genes with 130 co-expression events formed three bigger co-expression sub-networks (12 to 14 nodes) and 11 small sub-networks (two to five nodes). In pear, 293 LRR-RLK genes with 735 edges formed the main sub-network (182 nodes) and 24 small sub-networks (two to 30 nodes). In mei, 197 LRR-RLK genes with 1515 co-expression events formed the main sub-network (165 nodes) and 11 small sub-networks (two to eight nodes). In peach, 161 LRR-RLK genes with 1085 co-expression events formed two bigger sub-networks (57 and 49 nodes) and 17 small sub-networks (two to five nodes). The frequency distribution of co-expression events for each gene showed that some genes are more highly connected, exhibiting a higher number of edges, while others are less connected. These genes could be considered as hubs with importance in plant growth, development, and stress signaling. Comparing the top connected genes in each species revealed that genes in the subfamily XI-1 were in four of the five Rosaceae species. Specifically, mrna22117.1 (subfamily XI-1) and mrna23069.1 (subfamily I-1) for strawberry (number of edges = 2), MDP0000284907 (subfamily III), MDP0000166578 (subfamily XI-1), MDP0000309850 (subfamily XII-1) and MDP0000229275 (subfamily XI-1) for apple (number of edges = 10), Pm019513 (subfamily XI-1) for mei (number of edges = 46), ppa023423m (subfamily XII-1) for peach (number of edges = 38) and Pbr002506.1 (subfamily XI-1) for Chinese white pear (number of edges = 17).

### Tissue-specific expression and subcellular localization of Chinese white pear LRR-RLK genes

**Fig. 7:**
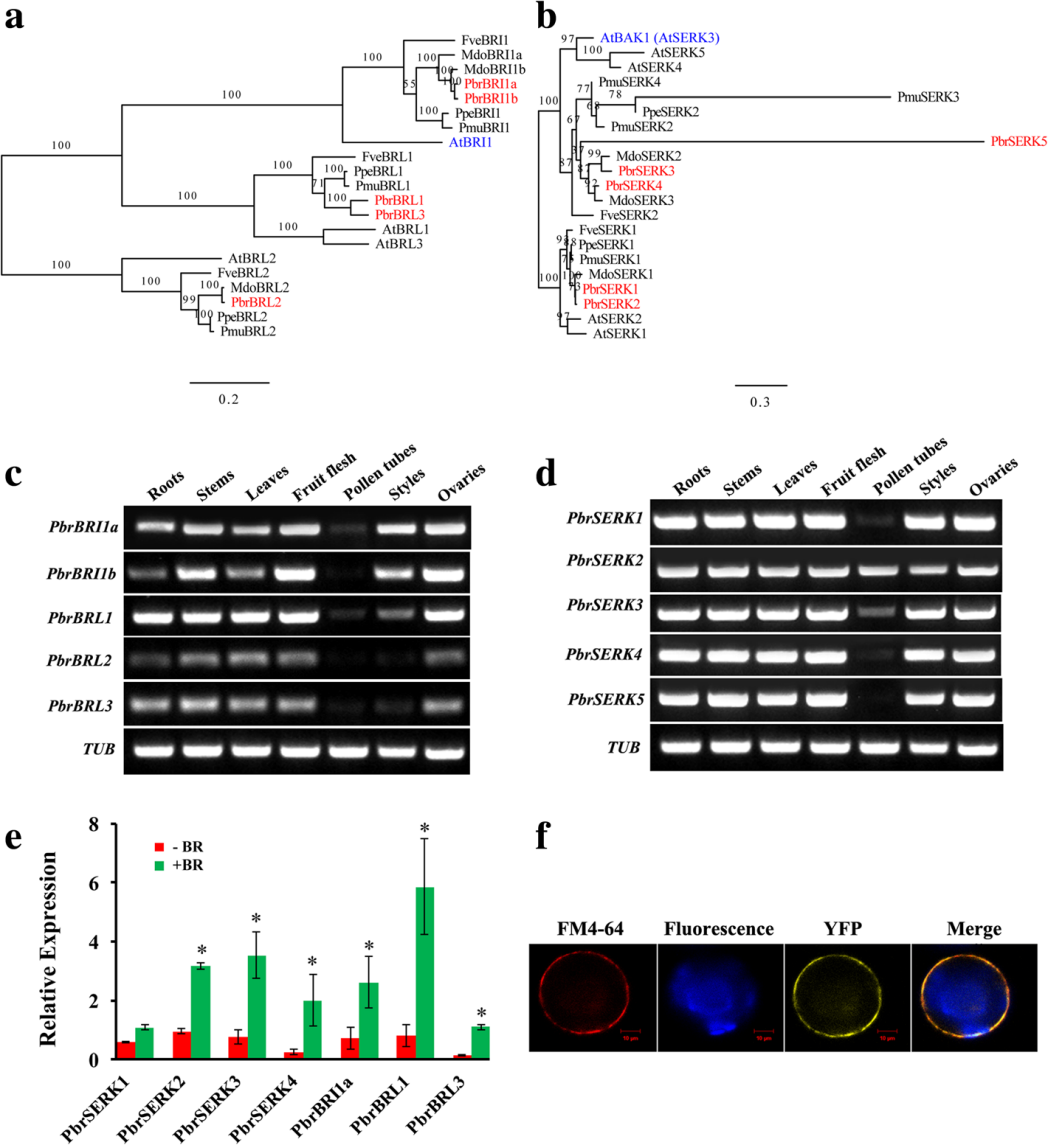
Phylogenetic trees, tissue-specific expression, and subcellular localization for the pear *BRI1*/*BRL* and *SERK* genes. **a** The phylogenetic tree of the *BRI1*/*BRL* genes identified in *Arabidopsis* and Rosaceae. **b** The phylogenetic tree of the *BAK1/SERK* genes identified in *Arabidopsis* and Rosaceae. **c** Tissue-specific expression of pear *BRI1*/*BRL* genes. **d** Tissue-specific expression of pear *SERK* genes. **e** The expression of seven selected pear *BRI1*/*BRL* and *SERK* genes in pollen tubes before and after BR treatment. All these seven genes expression has increased after exogenous BR treatment. **f** Subcellular localization analysis of *PbrSERK2* in the membranes of the *Arabidopsis* mesophyll protoplasts. YFP and FM4–64 fluorescence were localized exclusively to the membranes. Bar = 2 μm

*AtBRI1* and *AtBAK1* (also named *AtSERK3*) play key regulatory roles in BR signaling processes in *Arabidopsis*. In Chinese white pear, we identified two *BRI1* orthologs (*PbrBRI1a* and *PbrBRI1b*), three *BRI1-Like* (*BRL*) genes (*PbrBRL1*, *PbrBRL2* and *PbrBRL3*, orthologs of *AtBRL1*, *AtBRL2* and *AtBRL3*) (Fig. 7a) and five *Somatic Embryogenesis Receptor-like Kinase* (*SERK*) genes (Fig. 7b) that belong to the Xb-1 and II subfamilies, respectively. To study the gene regulatory network that controls pollen tube growth, expression pattern analyses of these genes were performed using reverse transcriptase PCR (RT-PCR) with RNA isolated from seven tissues (roots, stems, leaves, fruit flesh, pollen tubes, styles and ovaries) of *Pyrus bretschneideri* cv. Dangshansuli as shown in Fig. 7c–d. In roots, stems, leaves, fruit flesh, styles and ovaries, the expression of all *PbrBRLs* and *PbrSERKs* was detected. However, in pollen tubes, only three pear *BRI1/BRLs* genes (*PbrBRI1a*, *PbrBRL1* and *PbrBRL3*) and four *PbrSERKs* (*PbrSERK1*, *2*, *3*, and *4*) were expressed. Specifically, *PbrSERK2* was expressed in a very higher level compared with other *PbrSERKs*. Based on the results of RT-PCR, we tested the seven expressed genes (*PbrBRI1a*, *PbrBRL1*, and *PbrBRL3*; *PbrSERK1*, *2*, *3*, and *4*) for BR treatment experiment. The results revealed that exogenous BR could significantly increase the expression of these gene (Fig. 7e). Then, we tested the subcellular localization of *PbrSERK2* by the overexpression of 35S::*PbrSERK2*-YFP in *Arabidopsis* leaf protoplast stained with the FM4–64 dye were used as a positive contrast for cytoplasmic membrane. We found the extensive accumulation of yellow fluorescent protein (YFP) signals and red fluorescent (FM4–64) signals overlapping at the cell membrane (Fig. 7f), indicating that *PbrSERK2* was located on the cell membrane. These results reconfirmed the accuracy and reliability of the methods of this study and indicated that *PbrSERK2* might participate in BR signaling pathway in Chinese white pear.

## Discussion

In previous research, RLK genes were divided into six groups according to differences in the structures of their RLK extracellular domain, as follows: LRR [30–32], pathogenesis-related protein 5-like receptor kinase (PR5K) [33, 34], epidermal growth factor-like repeats (EGF) [35, 36], lectin-binding domain (LB) [37, 38], tumor necrosis factor receptor-like (TNFR) [39, 40], and S-domain [3, 41]. LRR-RLKs constitute more than half of the total number of RLKs. This group is one of the largest gene families in plants, playing important roles in plant growth and responses to pathogens [42–44]. In this study, we identified 1397 LRR-RLK genes in Rosaceae; of these, 201 genes were in strawberry, 244 in apple, 427 in Chinese white pear, 267 in mei, and 258 in peach (Table 2). These numbers are far greater than the average gene family size, which ranges from 1.71 to 2.17 in *Arabidopsis*, Chinese white pear, apple, and strawberry [20]. It was also noted that strawberry, apple, Chinese white pear, mei, and peach LRR-RLK genes represented 23.5%, 22.8%, 26.5%, 24.1%, and 25.2% of their total typical PK genes, respectively. These proportions are all higher than that in *Arabidopsis* (22.3%). In a previous study, it was asserted that LRR-RLK gene family expansion is mainly a consequence of high rates of duplicate retention [45], and that segmental and tandem duplication are two major sources of this expansion. In this study, we found that approximately one-third to two-thirds of LRR-RLK genes in Rosaceae could be explained by segmental duplication or tandem duplication, confirming this hypothesis. Considering the high ratio of LRR-RLK genes compared to PK genes in Rosaceae, the expansion of LRR-RLK genes could partially explain why the PK superfamily is larger in these species than in *Arabidopsis*. In Rosaceae, the Pyreae tribe was reported to have undergone one recent duplication [46] and it was found that 57% of apple LRR-RLK genes attributed to segmental duplication could be explained by this recent duplication, while the corresponding proportion was 54% in Chinese white pear. This explains why the proportions of total segmental duplicated genes in the Pyreae tribe are higher than in other studied species. We also noted that the genome and proteome sizes in the Pyreae tribe are larger than in other studied species, which could be another reason for the larger gene family size of LRR-RLK in these species. However, there is one exception to this in the apple genome, where the number of LRR-RLK genes identified were much less than in Chinese white pear, because we used the apple genome version 1.0 pseudo-haplotype assembly primary resource in the Genome Database for Rosaceae (GDR) [47]. The total number of genes in this version of the apple genome is 30,294, which is approximately half of that in the apple genome version 1.0. Nevertheless, we still observed a higher proportion of genes attributed to recent duplication.

Novel gene functions are acquired in four processes of genetic evolution: chromosome doubling, chromosome fragment insertion mutation, tandem duplication, and transposition [19, 48, 49]. Duplications can help species resist stress and to adapt to diverse environments by providing abundant genetic materials for evolution and producing numerous genetic variations. As described above, duplications could have driven the lineage-specific expansion of the LRR-RLK gene family, especially in the Pyreae tribe. We also noted that duplications had different effects on specific subfamilies, especially for tandem duplications. For instance, in *Arabidopsis*, 37 genes assigned to tandem duplication arrays were distributed in only five subfamilies, which represented approximately 16% of the 225 LRR-RLK genes; in addition, in Chinese white pear, 67 genes assigned to tandem duplication arrays were distributed in six subfamilies. Furthermore, the proportions of genes in tandem duplication arrays against the total LRR-RLK genes in each species in Rosaceae were not consistent, from 12% in strawberry and 13% in apple, less than in *Arabidopsis*, to 16% in Chinese white pear, 19% in mei, and 24% in peach, greater than or equal to that in *Arabidopsis* (Additional file 1: Table S10). Although different species had different numbers of subfamilies that expanded through tandem duplication, the observation that some subfamilies were consistently expanded through tandem duplication in all studied species (I-1, XI-1, and XII-1) while others were not indicates the preference for tandem duplication. Those subfamilies that expanded markedly through tandem duplication were partly consistent with those subfamilies with the greatest number of members, except subfamily III, which showed tandem duplication events only in apple. In contrast, the expansion of subfamily III was mainly the consequence of segmental duplication.

Many LRR-RLKs have been reported to play roles in plant disease resistance. For example, dependent on the suppressor of BIR1–1 (SOBIR1), *BAK1* negatively regulated *Arabidopsis* defenses interacted with the RLK *BIR1* (BAK1-interacting receptor-like kinase 1) [50]. Moreover, the steroidal hormones BRs were shown to promote pollen tube growth in *Arabidopsis* [51]. Interaction of *BRI1* with *BAK1* also play a key role in this promoted signaling network. In addition, *BAK1* acts not only as the second receptor of BRs, but is also a co-receptor of LRR-RLK FLS2 and EF-Tu receptor (EFR) [42, 52, 53]. In the processes of plant immune response, BAK1 and FLS2/EFR form a dynamic receptor and work with Botrytis-induced kinase 1 (BIK1). As a member of the *Catharanthus roseus* receptor-like kinase (CrRLK) family, FERONIA (FER) plays two important roles. First, it works as a pollen tube guidance protein during plant fertilization. Second, it plays a negative role in resistance to powdery mildew fungus [54–56]. The functionally redundant genes *ANXUR* (*ANX1* and *ANX2*) are close homologs of *FER* and expressed in pollen. Overexpressed *ANXUR* could negatively regulate pollen tube growth. However, more research is required to determine whether FER and ANXUR are involved in the process of BR promotion of pollen tube growth and plant immune responses and what roles they play. Although LRR-RLKs and CrRLKs, two important subfamilies of the RLK family, have evolved in different directions, they retain many similar biological functions in the process of plant growth.

According to the analyses of expression data presented in this work, we found that genes in the same subfamily have no consistent expression patterns; some genes in a subfamily might be highly expressed, whereas others are expressed at a low level or not at all. This pattern differs from an analysis of the soybean kinome, where it was found that gene members belonging to the same PK subfamilies showed similar expression patterns [57]. The reason for this difference could be the limitation of available expression data for soybean PKs, just 38%. By examining the expression of some duplicated genes within a subfamily, we found different expression patterns, which could be the result of the sub-functionalization or neo-functionalization of duplicated genes. Considering that some LRR-RLK subfamilies are very large, with over 100 members, the use of averaged expression data for such subfamilies could result in the loss of substantial information. LRR-RLK genes have been proven to have various functions in multiple tissues. Previous studies showed that LRR-RLK genes play important roles in root development; for example, *OsRPK1* in rice was shown to affect the root system architecture by negatively regulating polar auxin transport [58]. In this study, tissue expression analyses also showed that LRR-RLK genes were relatively highly expressed in root and stem compared with their levels in other examined tissues.

In our study, subfamily XI-1 was not only the largest subfamily, but was also highly expressed in all five Rosaceae species. It is worth mentioning that a total of 80 tandem duplicated genes were found only in the XI-1 subfamily, representing approximately 33.9% of the 236 tandem duplicated genes in the five Rosaceae species (Additional file 1: Table S10). This indicates that the gene expansion of this subfamily mainly occurred through fragment replication and tandem copies. CLAVATA (CLV), a member of the XI-1 subfamily in *Arabidopsis*, plays an important role in maintaining stem cell numbers in the apical meristem. *CLV1* encodes a typical LRR-RLK domain, the mutation of which leads to increases in the stem cell number of shoot apical meristem, flower number, and stem tip size [59]. Another example is provided by the LRR receptor kinase HAESA (HAE), which belongs to the XI-1 subfamily and is expressed in sepals, petals, the abscission zone of stamens, the bottom of the peduncle, and the base of the petiole. Decreased expression of this gene delays the withering of flower organs [60]. These results indicate that the Rosaceae XI-1 subfamily plays a very important role in growth and development. However, the functions of numerous LRR-RLK genes still remain unclear.

According to the expression analysis results presented here, we found that some genes from the III, V, VI-2, and LRR-XII-1 subfamilies were highly expressed during Chinese white pear pollen growth. Interestingly, a pattern was identified of most of these genes being up-regulated in the SPT stage, while some LRR-RLK genes were shown to promote pollen tube growth. Therefore, these highly expressed genes may function in the process of plant sperm cell release. In addition, there were 40 tandem duplicated genes in the XII-1 subfamily, which represented approximately 60% of the 67 tandem duplicated genes in Chinese white pear. This suggested that the gene tandem copies are one of the main causes of high expression of the XII-1 subfamily in pollen. Notably, the expression data showed that the Xb-1 and II subfamilies were not highly expressed in Chinese white pear pollen, but the RT-PCR experiment revealed that the genes *PbrBRL2* and *PbrSERK2*, which belong to these two subfamilies, were highly expressed in pollen tissues. This features might be associated with the age, location, and biotic and abiotic stresses of the tested samples.

## Conclusions

Our analyses of LRR-RLK genes in five Rosaceae species revealed their diversity in terms of member number, gene structure, tissue expression patterns, and expression patterns in response to stress. Expression patterns of these LRR-RLK genes should promote our understanding of the involvement of this gene family in plant growth, development, signal transduction, immunity, and stress responses.

## Methods

### Identification and classification of LRR-RLK genes

LRR-RLK genes were identified in five Rosaceae species: Chinese white pear (*Pyrus bretschneideri*), apple (*Malus domestica*), peach (*Prunus persica*), mei (*Prunus mume*), and strawberry (*Fragaria vesca*). To confirm the reliability of the identification process, which is similar to that used by [6], the model eudicot plant, *Arabidopsis*, was used as a reference, including in the identification process. Genome resources of the Chinese white pear were obtained from GigaDB (http://gigadb.org/dataset/100083). Genome resources of *Arabidopsis*, strawberry, and peach were obtained from Phytozome version 9.0 (http://phytozome.jgi.doe.gov/). Genome resources of mei (version 1.0) were obtained from the *Prunus mume* Genome Project website (https://github.com/lileiting/prunusmumegenome). Genome resources of apple (version 1.0 primary) were obtained from GDR (https://www.rosaceae.org). A summary of the genome information of *Arabidopsis* and the five Rosaceae species is provided in Table 1.

To identify LRR-RLK genes, putative PKs were initially obtained by searching hidden Markov models of the “typical” Pkinase clan [Pkinase (PF00069) and Pkinase_Tyr (PF07714)], obtained from the Pfam database version 28.0 [61], against the proteome in each studied species using HMMER v3.1 [62] with an E-value cut-off of 0.0001. After initial screening, typical PKs were identified with coverage of Pfam domain models of at least 50% [6]; otherwise, genes were designated as atypical PKs and excluded from further analysis. Furthermore, previously defined HMMs of different PK subfamilies (https://github.com/lileiting/Plant_Pkinase_fam.hmm) [6] were used to classify the identified typical PKs into families and subfamilies (genes were assigned to their best matched subfamily HMMs with an E-value cutoff of 0.0001). The subfamily HMMs were built from four plant model species [*A. thaliana* (dicotyledon), *Oryza sativa* (monocotyledon), *Physcomitrella patens* (moss), *Chlamydomonas reinhardtii* (a green alga)] with a phylogenetic approach as described by Lehti-Shiu and Shiu [6]. Briefly, a maximum likelihood phylogenetic tree for protein kinases of the four plant models and nine well-annotated eukaryote species (*Caenorhabiditis elegans*, *Dictyostelium discoideum*, *Drosophila melanogaster*, *Homo sapiens*, *Monosiga brevicollis*, *Mus musculus*, *Saccharomyces cerevisiae*, *Strongylocentrotus purpuratus* and *Tetrahymena thermophila*) was constructed and used for the classification.

To confirm this classification, a maximum likelihood phylogenetic tree was constructed with full-length protein sequences using RAxML [63]. Multiple sequence alignment of full-length protein sequences was performed using MUSCLE software [64]. Furthermore, phylogenetic trees for genes in subfamilies II and Xb-1 (fewer than 100 genes for each subfamily) were constructed using RAxML software [63]. The number of bootstrap replicates was automatically determined by the bootstrapping option provided by RAxML with the parameter “-N autoMRE” [65]. The online tool iTol [66] and Figtree software (version 1.4.2, http://tree.bio.ed.ac.uk/) were used to visualize the phylogenetic tree.

### Conserved domains and intron number

Conserved domains for each gene were obtained by scanning against the Pfam database version 28.0 using HMMER v3.1. Results were filtered with coverage for each domain of not less than 50%, and overlapping domains were resolved by comparing their E-values and retaining the best-matched domain. Intron number and intron length were calculated based on the gene feature files from the genome resources. Statistical analysis was performed using R programming language [67].

### Chromosome location and synteny analysis of Chinese white pear LRR-RLK genes

Chromosome locations of each LRR-RLK gene were obtained from their genome resources. Genome regions that showed synteny relationships were identified using the Multiple Collinearity Scan Toolkit (MCScanX) [68] with an E-value of 1e^−5^. Synonymous and non-synonymous rates were calculated using the “add_ka_and_ks_to_collinearity.pl” script in the MCScanX package. Tandem repeated genes were searched by comparing LRR-RLK genes in their corresponding positions in chromosome/scaffold sequences, and adjacent genes were designated as tandem duplicated genes.

### Expression analysis

To analyze the expression patterns of LRR-RLK genes in the five species, transcriptome or RNAseq data were retrieved from the NCBI SRA database [69], and accession numbers for these data are presented in the data availability section. In total, 13 datasets for Chinese white pear, 19 for apple, seven for peach, ten for mei, and 59 for strawberry were selected for expression analysis. The data retrieved from the SRA database were first decompressed into the FASTQ format using the SRA toolkit [69]. Each dataset was first mapped to its corresponding template genome using Tophat v2.1.0 [70] with default parameters; then, the expression level was normalized to fragments per kilobase of exon per million reads (FPKM) using Cufflinks v2.2.1 with the default parameters [71]. Additionally, the best matched *Arabidopsis* gene for each Rosaceae LRR-RLK genes were obtained using BLASTP [72] with E-value cutoff of 1e-5 and the function annotation for the *Arabiodpsis* genes were performed using R programming language [67].

### Construction of co-expression networks

Co-expression networks were generated for each of the five Rosaceae species for all identified members of the LRR-RLK gene family using the expression data analyzed above. The Pearson correlation coefficient (PCC) of FPKM values for each gene pair were calculated using R programming language [67]. The PCC threshold were 0.95 for all species. Co-expression network was visualized using Cytoscape v3.3.0 and analyzed using the NetworkAnalyzer plugin in Cytoscape [73].

### RT-PCR, qRT-PCR and subcellular localization

#### RT-PCR

Total RNA was extracted from seven *Pyrus bretschneideri* tissues (roots, stems, fruit flesh, pollen tubes, styles, and ovaries) using a total RNA extraction kit (Foregene, Chengdu, China). First-strand cDNA was synthesized using a reverse transcriptase kit (TaKaRa, Dalian, China). Actin was used as an internal control for RT-PCR. Amplifications were carried out with 35 cycles for all programs. The PCR products were detected with 1.5% agarose gel electrophoresis and visualized under UV light.

#### qRT-PCR

The mature ‘Dangshansuli’ (*Pyrus bretschneideri*) pollen was incubated in pollen germination medium (PGM) [74] at 25 °C for germination and growth with pollens and tubes collected after 0 h (mature pollen grain, MP), 40 min (hydrated pollen grain, HP), 6 h (growing pollen tube, PT) and 16 h (stopped-growth pollen tube, SPT). Additionally, the mature pollens were also incubated in PGM with the treatment of 0.01 μM epibrassinolide (epiBL; Sigma-Aldrich, E1641) at 25 °C after 3 h. Total RNA was then extracted for the synthesis of the first-strand cDNA. PCR reactions were carried out with 45 cycles using the SYBR Green Master Mix (SYBR Premix EX Taq™, TaKaRa). The pear tubulin (*TUB*) gene was used as the housekeeping gene for the normalization of the gene expression. The primer sequences used for amplifying the genes as shown in Fig. 6 are presented in Additional file 1: Table S17.

#### Subcellular localization

The 35S::*PbrSERK2-*YFP fusion constructs were infiltrated into *Arabidopsis* mesophyll protoplast [75]. After fusion, protoplasts were incubated at 25 °C for 24 h in the dark. After 24 h, 2 μM FM4–64 dye (T13320; Thermo Fisher Scientific) was added in the medium for 30 min to stain the cytoplasmic membrane of the protoplasts as positive control. The subcellular localization of the fused proteins was monitored using a confocal laser scanning microscope (LSM780; Zeiss, Oberkochen, Germany).

## Additional files

Additional file 1: Table S1. Typical protein kinase in *Arabidopsis and* Rosaceae. **Table S2.** Atypical protein kinase in *Arabidopsis* and Rosaceae. **Table S3.** Subfamily classification of protein kinases and their related information. **Table S4.** Conserved domains of LRR-RLK subfamily members in *Arabidopsis* and Rosaceae. **Table S5.** Statistics of domain numbers and list of conserved domains for LRR-RLK genes in *Arabidopsis* and Rosaceae. **Table S6.** Statistics of intron number and intron length for LRR-RLK genes in *Arabidopsis* and Rosaceae. **Table S7.** Collinearity events and Ka/Ks values LRR-RLK genes in *Arabidopsis* and Rosaceae. **Table S8.** Number of WGD events and number of genes involved in WGD events in 23 LRR-RLK subfamilies in *Arabidopsis* and Rosaceae. **Table S9.** List of tandem duplicated LRR-RLK genes in *Arabidopsis* and Rosaceae. **Table S10.** Number tandem arrays and tandem duplicated genes in 23 LRR-RLK subfamilies in *Arabidopsis* and Rosaceae. **Table S11.** Expression (FPKM) of 201 strawberry LRR-RLK genes. **Table S12.** Expression (FPKM) of 244 apple LRR-RLK genes. **Table S13.** Expression (FPKM) of 427 Chinese white pear LRR-RLK genes. **Table S14.** Expression (FPKM) of 267 mei LRR-RLK genes. **Table S15.** Expression (FPKM) of 258 peach LRR-RLK genes. **Table S16.** Co-expression network attributes of LRR-RLK genes in five Rosaceae species. **Table S17.** Sequences of primers used in qRT-PCR. (XLSX 1945 kb)

Additional file 2: Figure S1. Heat map of the expression patterns of 201 strawberry LRR-RLK genes in different tissues. Red and green colours correspond to up-regulation and down-regulation, respectively. Normalized gene expression values are provided in Additional file 1: Table S10. **Figure S2.** Heat map of the expression patterns of 244 apple LRR-RLK genes in different tissues. Red and green colours correspond to up-regulation and down-regulation, respectively. Normalized gene expression values are provided in Additional file 1: Table S11. **Figure S3.** Heat map of the expression patterns of 427 Chinese white pear LRR-RLK genes in different tissues. Red and green colours correspond to up-regulation and down-regulation, respectively. Normalized gene expression values are provided in Additional file 1: Table S12. **Figure S4.** Heat map of the expression patterns of 267 mei LRR-RLK genes in different tissues. Red and green colours correspond to up-regulation and down-regulation, respectively. Normalized gene expression values are provided in Additional file 1: Table S13. **Figure S5.** Heat map of the expression patterns of 258 peach LRR-RLK genes in different tissues. Red and green colours correspond to up-regulation and down-regulation, respectively. Normalized gene expression values are provided in Additional file 1: Table S14. **Figure S6.** Co-expression network of strawberry LRR-RLK genes. Nodes indicate genes and edges indicate significant co-expression events between genes. **Figure S7.** Co-expression network of apple LRR-RLK genes. Nodes indicate genes and edges indicate significant co-expression events between genes. **Figure S8.** Co-expression network of Chinese white pear LRR-RLK genes. Nodes indicate genes and edges indicate significant co-expression events between genes. **Figure S9.** Co-expression network of mei LRR-RLK genes. Nodes indicate genes and edges indicate significant co-expression events between genes. **Figure S10.** Co-expression network of peach LRR-RLK genes. Nodes indicate genes and edges indicate significant co-expression events between genes. (PDF 1698 kb)

## Abbreviations

BAK1: BRI1-associated kinase 1
BIK1: Botrytis-induced kinase 1
BIR1: BAK1-interacting receptor-like kinase 1
BR: Brassinosteroid
BRI1: Brassinosteroid-insensitive 1
BRL: BRI1-Like
CLV: CLAVATA
CrRLKs: *Catharanthus roseus* receptor-like kinases
EFR: EF-Tu receptor
EGF: Epidermal growth factor-like repeats
FER: FERONIA
FLS2: Flagellin-sensitive 2
FPKM: Fragments per kilobases exons million reads
GDR: Database for Rosaceae
HAE: HAESA
HMM: Hidden Markov models
HP: Hydrated pollen grains
JA: Jasmonic acid
K_s_: Synonymous substitution rate
LB: Lectin-binding domain
LRR_8: Leucine rich repeat 8
LRRNT_2: Leucine rich repeat N-terminal 2
LRR-RLK: Leucine-rich repeat receptor-like kinases
MCScanX: Multiple Collinearity Scan Toolkit
MP: Mature pollen grains
MYA: Million years ago
NCBI: National Center for Biotechnology Information
PCC: Pearson correlation coefficient
Pfam: Protein family (http://pfam.xfam.org)
PK: Protein kinase
PR5K: Pathogenesis-related protein 5-like receptor kinase
PT: Growing pollen tubes
*p*-value: Probability value
RLK: Receptor-like kinase
RNA-seq: RNA sequencing
RPK1: Receptor-like protein kinase 1
RT-PCR: Reverse transcriptase PCR
SERK: *Somatic Embryogenesis Receptor-like Kinase*
SOBIR1: Suppressor of BIR1–1
SPT: Stopped-growth pollen tubes
SRA: Sequence Read Archive
TNFR: Tumor necrosis factor receptor-like
WGD: Whole genome duplication
XII-1: RLK-Pelle_LRR-XII-1
YFP: Yellow fluorescent protein
