# Supplementary material for: Genome-wide characterization, evolution, and expression analysis of the leucine-rich repeat receptor-like protein kinase (LRR-RLK) gene family in Rosaceae genomes
Source: BMC Genomics. 2017 Oct 10;18:763. doi: 10.1186/s12864-017-4155-y (PMC5635495; doi:10.1186/s12864-017-4155-y)
Supplement: Supplementary file 2 — Heat map of the expression patterns of 201 strawberry LRR-RLK genes in different tissues. Red and green colours correspond to up-regulation and down-regulation, respectively. Normalized gene expression values are provided in Additional file 1: Table S10. Figure S2. Heat map of the expression patterns of 244 apple LRR-RLK genes in different tissues. Red and green colours correspond to up-regulation and down-regulation, respectively. Normalized gene expression values are provided in Additional file 1: Table S11. Figure S3. Heat map of the expression patterns of 427 Chinese white pear LRR-RLK genes in different tissues. Red and green colours correspond to up-regulation and down-regulation, respectively. Normalized gene expression values are provided in Additional file 1: Table S12. Figure S4. Heat map of the expression patterns of 267 mei LRR-RLK genes in different tissues. Red and green colours correspond to up-regulation and down-regulation, respectively. Normalized gene expression values are provided in Additional file 1: Table S13. Figure S5. Heat map of the expression patterns of 258 peach LRR-RLK genes in different tissues. Red and green colours correspond to up-regulation and down-regulation, respectively. Normalized gene expression values are provided in Additional file 1: Table S14. Figure S6. Co-expression network of strawberry LRR-RLK genes. Nodes indicate genes and edges indicate significant co-expression events between genes. Figure S7. Co-expression network of apple LRR-RLK genes. Nodes indicate genes and edges indicate significant co-expression events between genes. Figure S8. Co-expression network of Chinese white pear LRR-RLK genes. Nodes indicate genes and edges indicate significant co-expression events between genes. Figure S9. Co-expression network of mei LRR-RLK genes. Nodes indicate genes and edges indicate significant co-expression events between genes. Figure S10. Co-expression network of peach LRR-RLK genes. Nodes indicate genes and edges indic [file 12864_2017_4155_MOESM2_ESM.pdf]

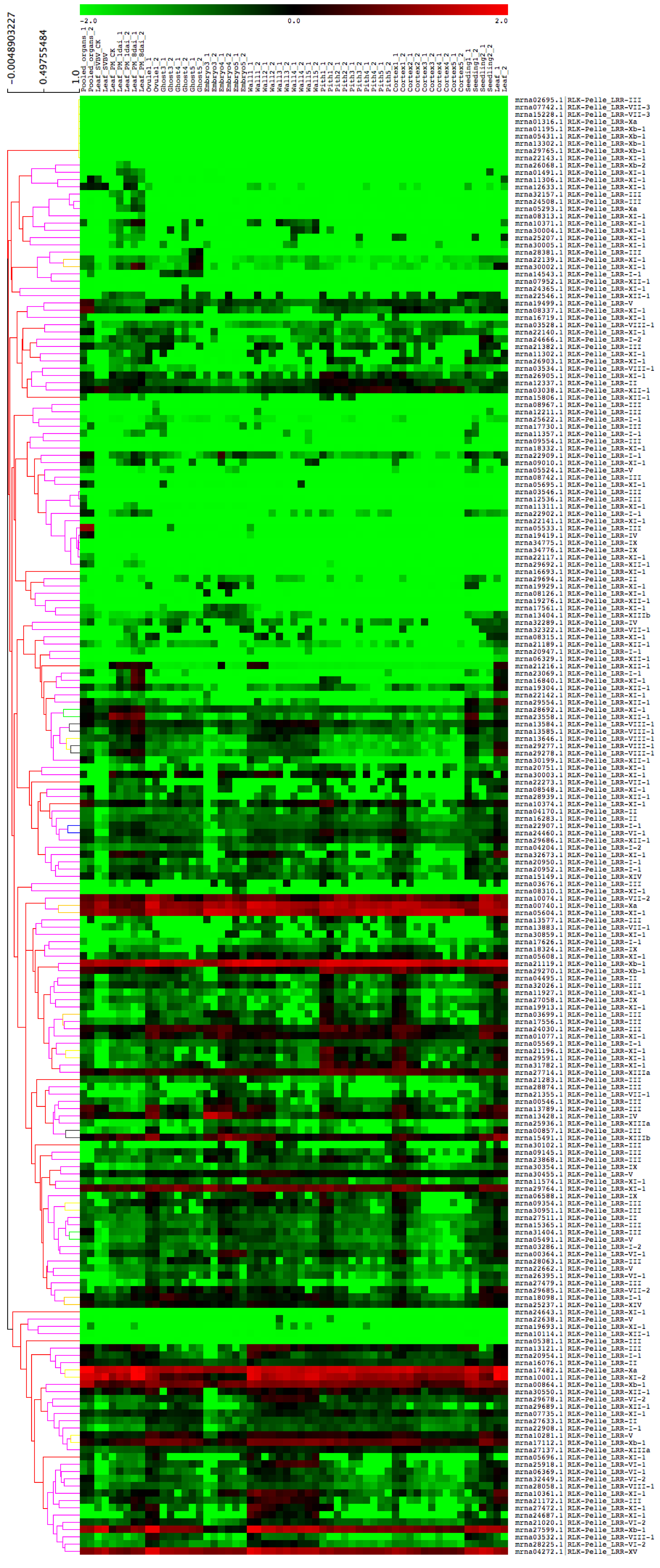


**Supplementary Figure 1. Heat map of the expression patterns of 201 strawberry LRR-RLK genes in different tissues. Red and green colors correspond to up-regulation and down-regulation, respectively. Normalized gene expression values are provided in Supplementary Table 10.**

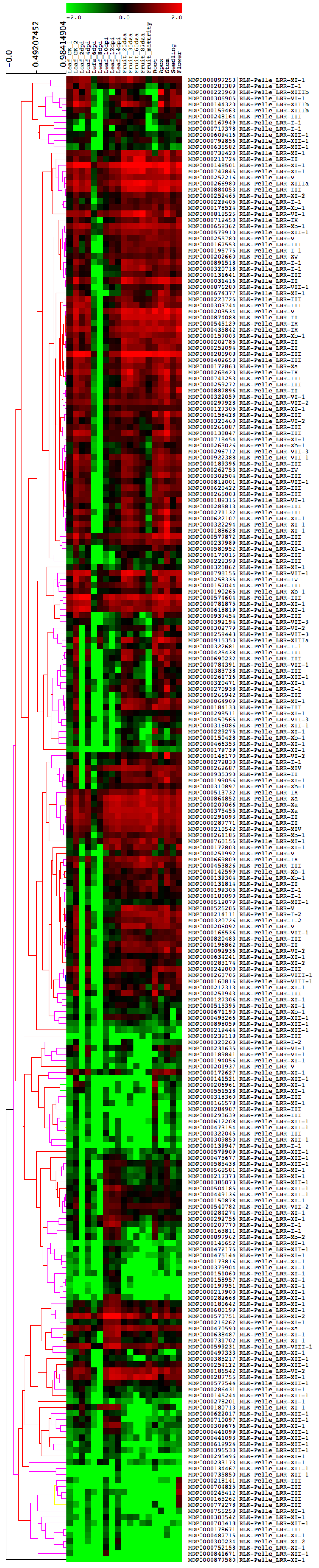


**Supplementary Figure 2. Heat map of the expression patterns of 244 apple LRR-RLK genes in different tissues. Red and green colors correspond to up-regulation and down-regulation, respectively. Normalized gene expression values are provided in Supplementary Table 11**

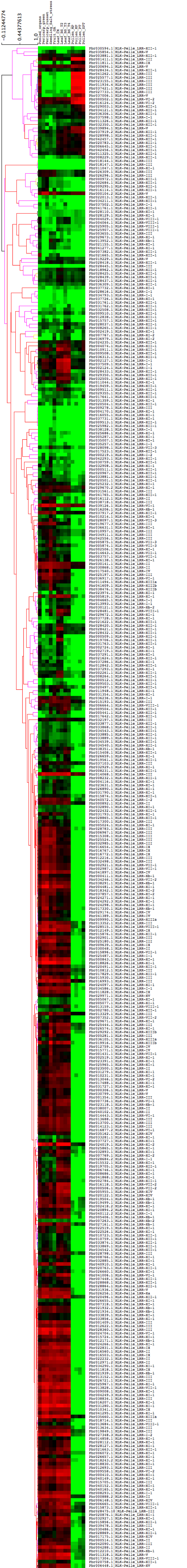


**Supplementary Figure 3. Heat map of the expression patterns of 427 Chinese white pear LRR-RLK genes in different tissues. Red and green colors correspond to up-regulation and down-regulation, respectively. Normalized gene expression values are provided in Supplementary Table 12.**

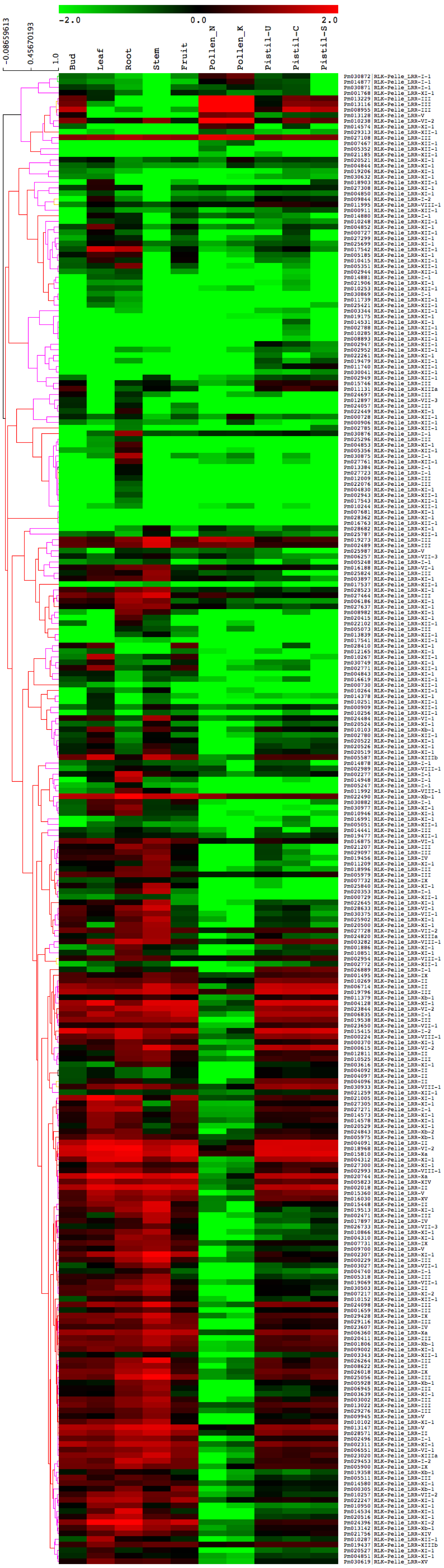


**Supplementtary Figure 4. Heat map of the expression patterns of 267 mei LRR-RLK genes in different tissues. Red and green colours correspond to up-regulation and down-regulation, respectively. Normalized gene expression values are provided in Supplementary Table 14.**

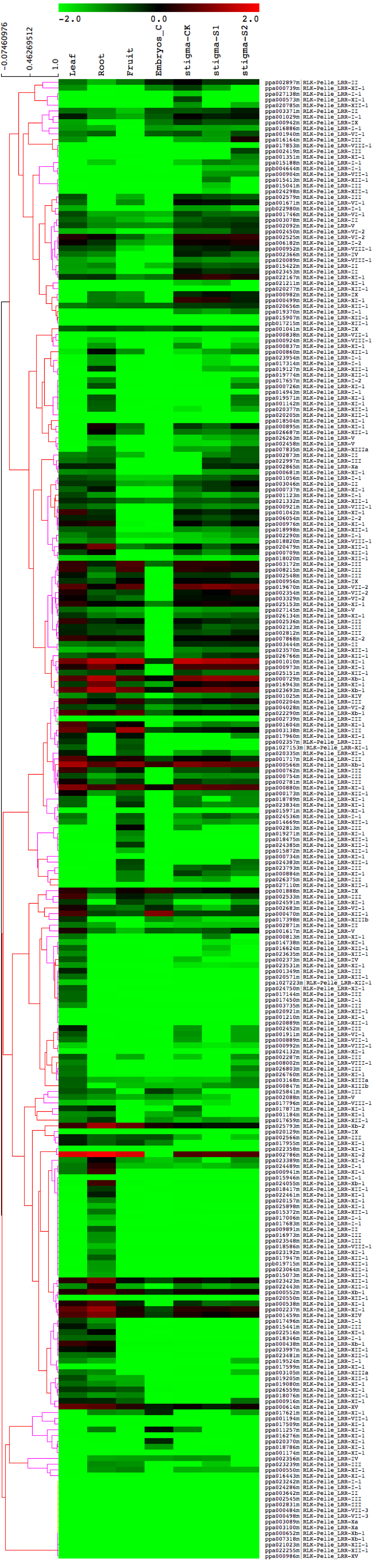


**Supplementary Figure 5. Heat map of the expression patterns of 258 peach LRR-RLK genes in different tissues. Red and green colours correspond to up-regulation and down-regulation, respectively. Normalized gene expression values are provided in Supplementary Table 15.**

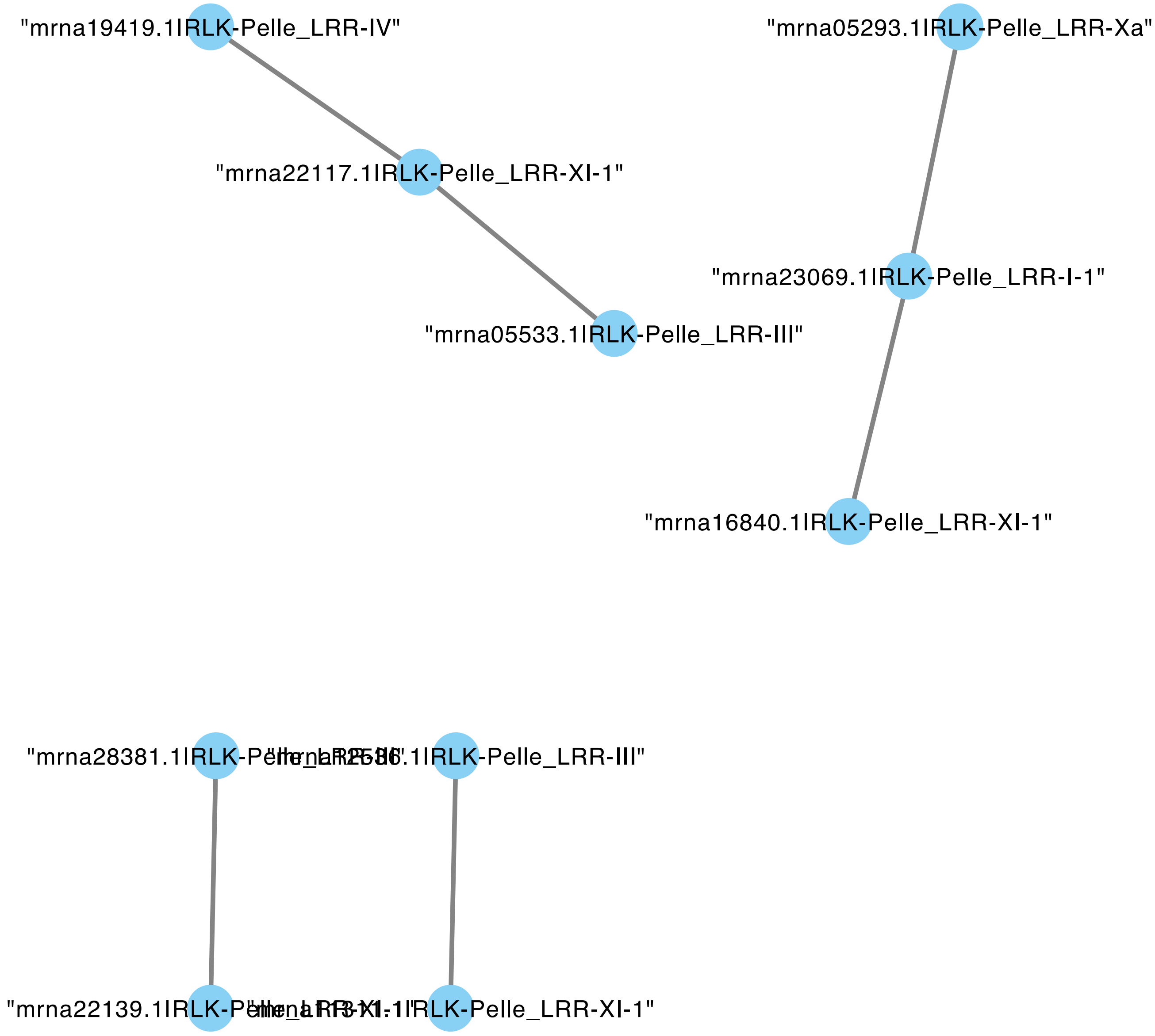


**Supplementary Fig. 6. Co-expression network of strawberry LRR-RLK genes. Nodes indicate genes and edges indicate significant co-expression events between genes.**

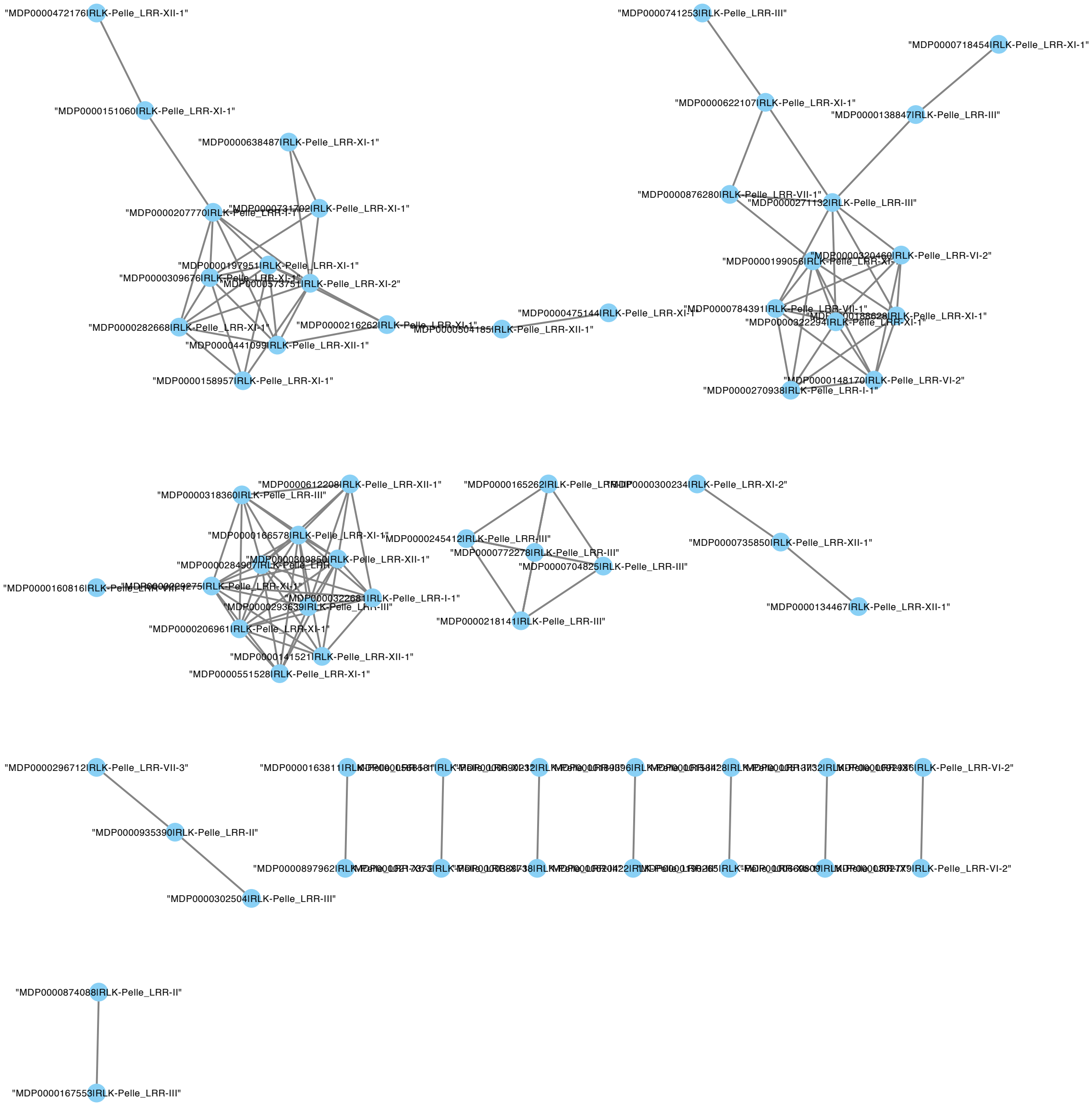


**Supplementary Fig. 7. Co-expression network of apple LRR-RLK genes. Nodes indicate genes and edges indicate significant co-expression events between genes.**

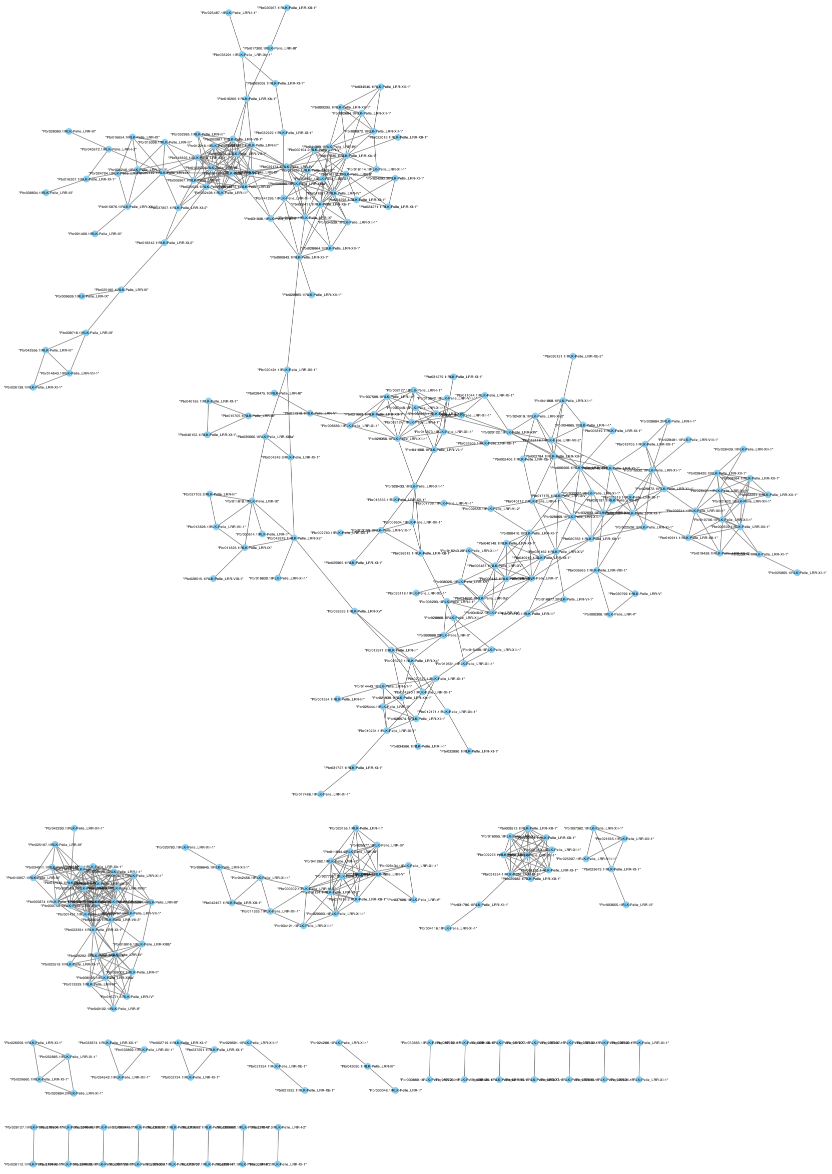

**Supplementary Fig. 8. Co-expression network of Chinese white pear LRR-RLK genes. Nodes indicate genes and edges indicate significant co-expression events between genes.**

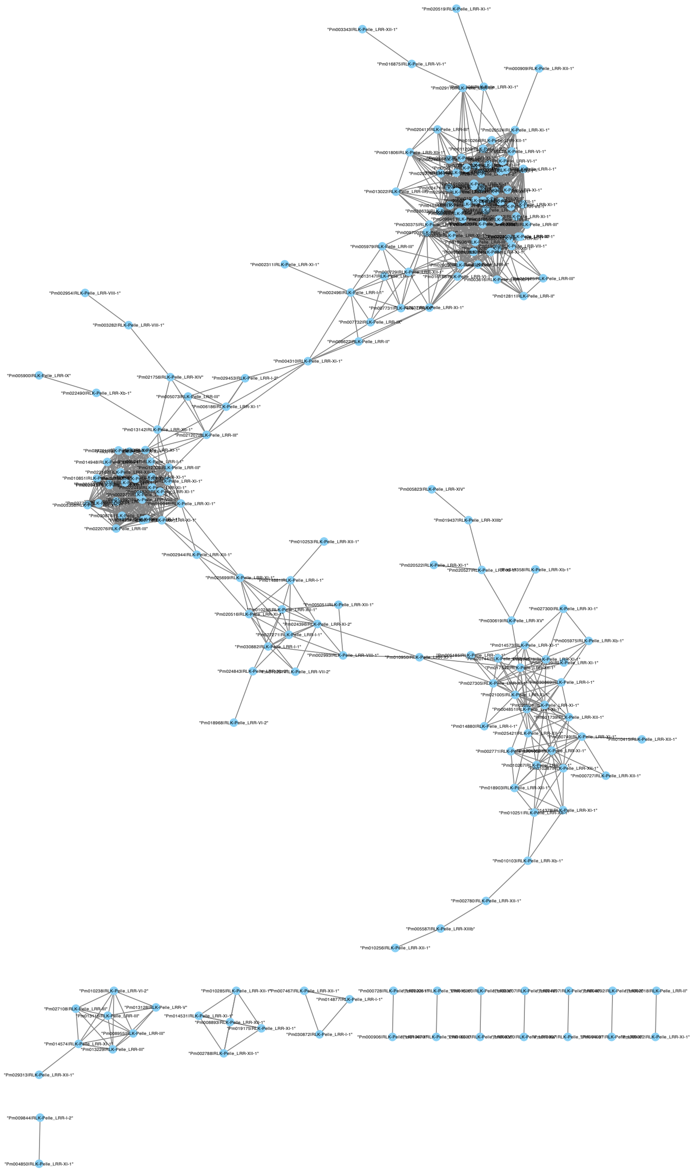

**Supplementary Fig. 9. Co-expression network of mei LRR-RLK genes. Nodes indicate genes and edges indicate significant co-expression events between genes.**

Supplementary Fig. 9. Co-expression network of mei LRR-RLK genes. Nodes indicate genes and edges indicate significant co-expression events between genes.

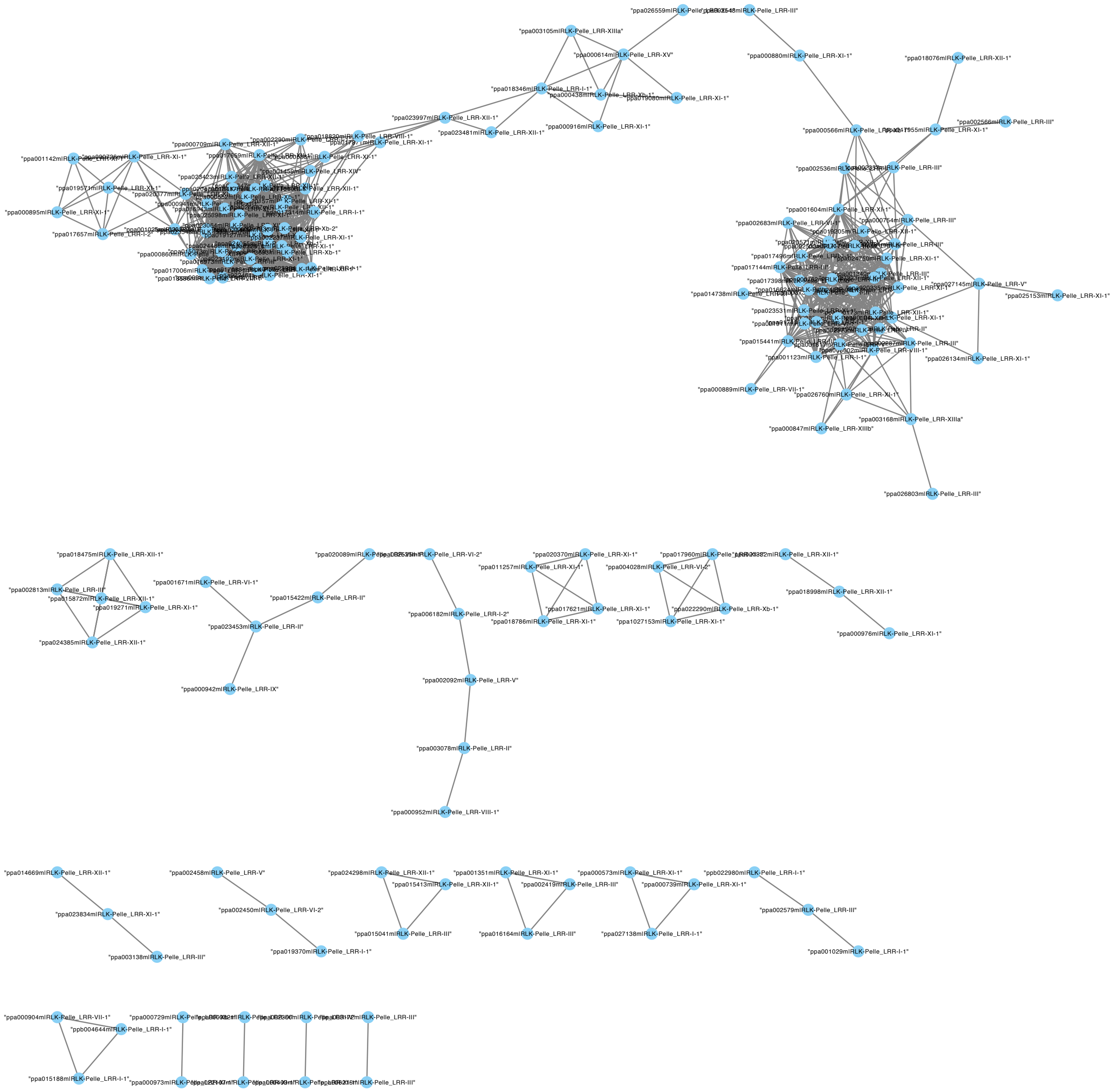

**Supplementary Fig. 10. Co-expression network of peach LRR-RLK genes. Nodes indicate genes and edges indicate significant co-expression events between genes.**
